# Editorial: Immune-mediated damage to the heart and lungs in autoimmune diseases

**DOI:** 10.3389/fimmu.2024.1407748

**Published:** 2024-04-05

**Authors:** Lingling Wei, Per Hydbring, Li Long

**Affiliations:** ^1^Center for Endocrine Metabolism and Immune Diseases, Beijing Luhe Hospital, Capital Medical University, Beijing, China; ^2^Department of Oncology-Pathology, Karolinska Institutet, Solna, Sweden; ^3^Department of Rheumatology and Immunology, Sichuan Academy of Medical Sciences and Sichuan Provincial People’s Hospital, University of Electronic Science and Technology of China, Chengdu, China; ^4^Clinical Immunology Translational Medicine Key Laboratory of Sichuan Province, Sichuan Provincial People’s Hospital, University of Electronic Science and Technology of China, Chengdu, China

**Keywords:** autoimmune disease, rheumatic immune diseases, cardiac, lung, immune-mediated damage

Autoimmune diseases are a collection of conditions where the immune system erroneously assaults healthy tissues and organs, causing inflammation and subsequent damage. Among these, the immune-mediated damage to the heart and lungs is a complex and pivotal field of research. Such injuries can lead to a wide array of severe health complications. Treating this damage poses significant challenges, often requiring strategies that aim to control inflammation, mitigate immune responses, and alleviate symptoms. This typically involves the administration of immunosuppressants, hormonal drugs, or other anti-inflammatory medications. As our understanding of these diseases deepens, researchers are exploring innovative treatment approaches, such as targeted therapies that precisely aim at specific immune pathways or molecules. It is anticipated that these novel methods will more accurately modulate immune responses, minimize side effects, and improve treatment outcomes. By meticulously studying the pathogenesis and treatment modalities of these diseases, we aspire to offer patients more effective therapeutic options and enhance their overall quality of life.

Four studies on this Research Topic have focused on different aspects of rheumatic immune diseases, including relapsing polychondritis (RP), rheumatoid arthritis (RA), sarcoidosis of the heart, and idiopathic pulmonary fibrosis (IPF) caused by dermatomyositis (DM). These studies have not only delved into the pathogenesis of these diseases, but also revealed the associations and interactions among different diseases, providing new perspectives and strategies for clinical diagnosis and treatment.

Firstly, the study conducted by Yin et al. revealed the clinical characteristics and related factors of cardiac involvement in patients with relapsing polychondritis (RP). Their research identifies a series of independent risk factors linked to cardiac involvement, including age, central nervous system (CNS) involvement, a neutrophil-to-lymphocyte ratio (NLR) exceeding 6.41, and a disease duration spanning over four years. This is crucial for accurately assessing risk and implementing early intervention strategies in RP patients. Notably, there are significant variations in the reported rates of system involvement in RP, with the comprehensive report by Japanese scholars mentioned in the article serving as a key reference. Additionally, many other reports tend to focus on individual cases of cardiac involvement in RP (1). Furthermore, the study introduces five distinct clinical patterns of RP, highlighting the mutual exclusivity between the cardio-cerebral pattern and the airway pattern, thereby enhancing our understanding of the complex and diverse nature of RP.

Furthermore, the study led by Zhao et al. reviewed the pathogenesis and therapeutic potential of Notch signaling in angiogenesis in rheumatoid arthritis (RA). They emphasized the role of stromal cells and adipokines in the angiogenic process and explored the impact of epigenetic regulation of Notch signaling on RA angiogenesis. This offers fresh perspectives on RA treatment, particularly the intriguing potential of targeting the Notch signaling pathway as an innovative therapeutic approach. By understanding the role of Notch signaling in RA angiogenesis, we can potentially develop more effective treatments that target the underlying mechanisms of the disease, leading to improved outcomes for RA patients. While some scholars have previously reviewed the role of the Notch signaling pathway in RA, comprehensively outlining its diverse functions (2), this article takes a further step by exploring its specific mechanism in regulating angiogenesis in RA and highlighting its potential therapeutic significance.

Thirdly, Frischknecht et al. evaluated the effectiveness and safety of various treatment regimens for cardiac sarcoidosis, utilizing 18F-fluorodeoxyglucose positron emission tomography/computed tomography (18F-FDG PET/CT). They found that tumor necrosis factor inhibitors (TNFi) remain effective and safe even in patients with severely reduced left ventricular ejection fraction. When compared to other treatment options, TNFi exhibited superior control over myocardial inflammation. This discovery offers a promising new treatment option for cardiac sarcoidosis, potentially improving the quality of life for patients. The study underscores the importance of personalized medicine and the need for further research to optimize treatment strategies for this complex condition. Two studies have also delved into the application of cardiac sarcoidosis and PET technology, but their research objectives, methods, and clinical applications vary. The study by Ron Blankstein et al. is instrumental in predicting disease progression earlier, thereby aiding doctors in devising more tailored treatment plans for patients (3). Furthermore, the study by Daniele Muser et al. focuses specifically on patients with certain clinical manifestations, such as ventricular tachycardia, providing novel methods and evidence for their prognostic assessment (4).

Finally, Zeng et al. analyzed the molecular mechanisms of idiopathic pulmonary fibrosis (IPF) caused by dermatomyositis (DM) using bioinformatics methods. They identified four hub genes and shared molecular pathways between DM and IPF, providing crucial clues for a deeper understanding of the complex mechanisms underlying these diseases. Additionally, these discoveries offer potential targets for diagnosis and therapeutic intervention, pointing to new directions for future research. Another study conducted by the same research team also centering on dermatomyositis, employed a combination of bioinformatic analysis and *in vivo* validation. However, the focus of this latter study was on mitochondrial-related hub genes in dermatomyositis (5). By approaching the pathogenesis of dermatomyositis from a different angle, they have provided novel insights and promising directions for future research and treatment strategies.

In summary, these studies have not only illuminated the intricate and multifaceted nature of rheumatic immune diseases but have also furnished us with profound insights and therapeutic strategies for addressing these conditions. As research continues to progress and technological advancements are made, we are optimistic that we will be able to better manage and treat these diseases, ultimately enhancing the quality of life for patients.

## Author contributions

LW: Data curation, Investigation, Writing – original draft, Writing – review & editing. PH: Writing – review & editing. LL: Writing – review & editing.
